# Visualization of Periplasmic and Cytoplasmic Proteins with a Self-Labeling Protein Tag

**DOI:** 10.1128/JB.00864-15

**Published:** 2016-03-17

**Authors:** Na Ke, Dirk Landgraf, Johan Paulsson, Mehmet Berkmen

**Affiliations:** a New England Biolabs, Ipswich, Massachusetts, USA; b Department of Systems Biology, Harvard Medical School, Boston, Massachusetts, USA

## Abstract

The use of fluorescent and luminescent proteins in visualizing proteins has become a powerful tool in understanding molecular and cellular processes within living organisms. This success has resulted in an ever-increasing demand for new and more versatile protein-labeling tools that permit light-based detection of proteins within living cells. In this report, we present data supporting the use of the self-labeling HaloTag protein as a light-emitting reporter for protein fusions within the model prokaryote Escherichia coli. We show that functional protein fusions of the HaloTag can be detected both *in vivo* and *in vitro* when expressed within the cytoplasmic or periplasmic compartments of E. coli. The capacity to visually detect proteins localized in various prokaryotic compartments expands today's molecular biologist toolbox and paves the path to new applications.

**IMPORTANCE** Visualizing proteins microscopically within living cells is important for understanding both the biology of cells and the role of proteins within living cells. Currently, the most common tool is green fluorescent protein (GFP). However, fluorescent proteins such as GFP have many limitations; therefore, the field of molecular biology is always in need of new tools to visualize proteins. In this paper, we demonstrate, for the first time, the use of HaloTag to visualize proteins in two different compartments within the model prokaryote Escherichia coli. The use of HaloTag as an additional tool to visualize proteins within prokaryotes increases our capacity to ask about and understand the role of proteins within living cells.

## INTRODUCTION

Microscopic visualization of proteins within living cells has increased our understanding of many biological processes. Visualization of proteins by fusing protein domains capable of producing fluorescent (e.g., green fluorescent protein [GFP]) or luminescent (e.g., luciferase [Luc]) light is currently the most powerful method to investigate biomolecules in their native context (1, 2). As would be expected, each light-producing protein domain has limitations in its spectral properties, depending on the *in vivo* conditions where it is expressed (3). This is further complicated when fusion protein domains, such as GFP or Luc, are required to cross membranes in order to be visualized in noncytoplasmic compartments. Therefore, it is essential to have myriad protein domain fusions that can permit the visualization of proteins within the context of a living cell.

Proteins targeted to noncytoplasmic compartments, such as the periplasmic compartment of Escherichia coli, need to cross the inner membrane. If the client protein is targeted to the periplasm via the Sec pathway, the client protein must remain unfolded in a secretion-competent state. Protein domains that fold quickly into stable structures can hinder secretion (4, 5). Even if the fusion protein remains secretion competent, its overexpression in the periplasm usually fails due to limitations in the secretion capacity of the cell and requires optimization of expression (5, 6). Furthering the difficulty of periplasmic expression of proteins are the porous nature of the outer membrane to reactive small chemicals and the presence of enzymes responsible for the oxidation of cysteines to form disulfide bonds (7). Taken together, proteins expressed in the periplasm must cross the inner membrane without overwhelming the secretion machinery and are prone to chemical insults, oxidation, and, therefore, misfolding (7).

Although expression of GFP in the cytoplasm of E. coli was first demonstrated in 1994 (8), the periplasmic expression of GFP fusion proteins remained problematic at first. Early attempts at expressing GFP fusion proteins using the signal sequence (ss) of MalE (ssMalE-GFP) failed, as functional GFP could not be detected in the periplasm (9). This was most likely due to misfolding and improper chromophore formation of GFP in the periplasm (10). A solution was reported a year later by targeting GFP to the periplasm via the signal sequence of the twin-arginine translocation (TAT) pathway (ssTorA-GFP), which can export cytoplasmically folded proteins (11). This was also the first demonstration of TAT-dependent export of an active heterologous protein. However, 50% of the ssTorA-GFP proteins failed to be exported and remained in the cytoplasm, which resulted in a relatively high cytoplasmic fluorescent signal (11). This problem was solved by fusing the 11-amino-acid SsrA tag to the C terminus of ssTorA-GFP, which caused degradation of all remaining cytoplasmic GFP-tagged molecules (12). As with GFP, protein mislocalization and nonuniform labeling of the bacterial cells were observed with ssTorA-yellow fluorescent protein (YFP) (13).

Significant progress in expressing fluorescent proteins in the periplasm resulted from the development of mRFP1, a red fluorescent protein derived from the tetramer DsRed of Discosoma coral, which was engineered such that the fluorescent species is a monomer (14). In E. coli, mRFP1 was used with success to visualize several proteins in the periplasm: the membrane-anchored protease MmpA, *sec*-secreted MBP, and cotranslocationally secreted DsbA (13). However, the fluorescent signal in the periplasm was significantly brighter for the signal recognition particle (SRP)-targeted DsbA-mRFP1 than for *sec*-targeted MBP-mRFP1, indicating that mRFP1 might have folding or secretion issues when it is targeted by a *sec*-dependent signal peptide, similar to what was observed for GFP and YFP. Finally, neither immunoblotting nor biochemical activities of the DsbA and MBP fusions were reported for the mRFP1 fusions.

Folding of wild-type (wt) GFP in E. coli is problematic and motivated the engineering of several GFP mutants, such as fluorescence-activated cell sorter (FACS)-optimized GFP (15), folding reporter GFP (16), enhanced GFP (EGFP) (17), and superfolder GFP (sfGFP) (18). Unlike previous versions of GFP, sfGFP was used with success to visualize proteins in the endoplasmic reticulum (ER) of eukaryotic cells (19) and the bacterial periplasm (20). However, as with previous observations, sfGFP could not be targeted to the periplasm via the *sec* pathway, and efficient secretion of sfGFP to the periplasm occurred only when it was targeted cotranslationally via the SRP pathway (20). In addition, molecular oxygen is essential for the posttranslational maturation of most fluorescent proteins, including all GFP- and DsRed-derived fluorescent proteins (21), limiting their use to aerobic conditions.

An alternative method of visualizing proteins in cells is with the use of luciferases, which can utilize chemical substrates to produce light. The first successful expression of luciferase in the periplasm of E. coli was achieved with firefly luciferase (Fluc) (22); consequently, fusions of Fluc have been used to study protein localization (23). However, the dependency of Fluc on ATP for light production limits its *in vivo* microscopic use in the periplasm, which lacks ATP. Luciferases that use coelenterazine and are not ATP dependent thus became an attractive alternative. The first ATP-independent luciferase to be expressed in the periplasm was Renilla luciferase (RLuc), which was then further engineered for increased stability (24). This engineered Renilla luciferase (RLuc8) utilizes coelenterazine and was expressed periplasmically to visualize Salmonella enterica serovar Typhimurium strains within a living mouse (25). Unfortunately, extensive characterization of periplasmically expressed RLuc8 has not been conducted to date. Similarly, several attempts of periplasmic expression of the luciferase from Gaussia princeps (Gluc) in E. coli have been reported, albeit with limited success and low yields (26–28), which thus limit its use for microscopy.

Recent additions to the repertoire of light-producing reporter proteins are the self-labeling systems, such as the SNAP tag (29), HaloTag (30), TMP tag (31), BL tag (32), and tetracysteine tag (33), and the enzyme-mediated systems, such as phosphopantetheinyl transferases (34), sortase (35), and lipoic acid ligase (36). These small protein domains can form covalent linkages to exogenously added fluorophores and allow microscopic investigations of the labeled fusion proteins. The HaloTag is a mutated 34-kDa dehalogenase from Rhodococcus rhodochrous that can covalently bind a diverse set of chloroalkane ligands (37). Its catalytic active-site histidine at position 272 has been mutated to phenylalanine, resulting in a mutant dehalogenase that forms a stable covalent bond with synthetic ligands, such as the fluorophore tetramethylrhodamine (TMR). HaloTags have been successfully used in eukaryotic cells to visualize proteins as a carboxyl terminus fusion in the cytoplasm (38), nucleus (39), and mitochondrial matrix (40) and in the endoplasmic reticulum of the cell (41). The use of self-labeling tags has several advantages over currently available fluorescent proteins, such as superior spectroscopic properties, the availability of an astonishing number of ligands (e.g., multiple fluorescent dyes, biotin, quantum dots, conjugated beads, DNA oligonucleotides, and ligand building blocks with a variety of functionalities) that can be used with a single fusion for various experimental purposes, and, in strong contrast to the situation with GFP and dyes, the lack of dependence on molecular oxygen for their chromophore formation. Even though the HaloTag (42) and SNAP tag (43) have been expressed with success as fusion proteins in E. coli, the use of the HaloTag thus far in visualizing proteins in the two compartments of bacteria has not been accomplished.

Here, we demonstrate the use of the HaloTag as a viable alternative to fluorescent protein tags and present a detailed characterization of its use in conducting microscopic studies of both periplasmic and cytoplasmic proteins.

## MATERIALS AND METHODS

### E. coli strains and plasmids.

Bacterial strains and plasmids used in this work are described in Table 1 and were constructed using standard molecular and genetic techniques (44). The primer sequences are listed in Table 2.

**TABLE 1:** Bacterial strains and plasmids utilized in this study

Strain or plasmid	Relevant genotype or description	Reference
Strains		
MB10	DHB4 F′ *lac pro lacI^q^* Δ(*malF*)*3* Δ(*phoA*)PvuII *phoR* Δ(*lac*)*X174* Δ(*ara-leu*)*7697 araD139 galE* (or *galU*) *galK*	62
MB68	MB10 Δ*dsbA*	63
MB3104	MB10 *dsbA*::Halo	This study
MB3105	MB10 *dsbA*::HaloSS	This study
MB3106	MB10 *dsbA*::sfGFP	This study
MB3769	MB10 pDSW204-ssDsbA-Halo	This study
MB3123	MC4100	43
MB4156	MB3123 Δ*rpoS*	This study
DHL708	MC4100 Δ*clpPX*	43
MB3128	MB3123 *clpP*::Halo	This study
MB3717	MB3123 *clpP*::msfGFP	This study
Plasmids		
pDSW204	Mutant *lacUV5* promoter, pBR322 origin, Amp^r^	64
pDSW204-ssDsbA-Halo	Signal sequence of DsbA fused to the HaloTag in pDSW204	This study
pDHL584	pUC19-linker-sfGFP-FRT-Kan^r^-FRT	43
pDHL940	pUC19-linker-HaloTag H7-FRT-Kan^r^-FRT	This study
pDHL1029	pUC19-linker-msfGFP-FRT-Kan^r^-FRT	This study
pUC57-HaloSS-Kan^r^	Cysteineless HaloTag mutant	This study
pDHL-HaloSS	pUC19-linker-HaloSS-FRT-Kan^r^-FRT	This study

**TABLE 2:** Primers used in this study

Primer	Sequence
DsbA_F	TTTTTGTTCAGCAGTATGCTGATACAGTGAAATATCTGTCCGAGAAAAAAAGCGGTGGCGGTGGCAGTAA
DsbA_R	AATAAAAAAAGCCCGTGAATATTCACGGGCTTTATGTAATTTACATTGAAATTCCGGGGATCCGTCGACC
DHL_P182_F	CTGAAGCGGTGGAATACGGTCTGGTCGATTCGATTCTGACCCATCGTAATAGCGGTGGCGGTGGCAGTAA
DHL_P183_R	AGCGTTGTGCCGCCCTGGATAAGTATAGCGGCACAGTTGCGCCTCTGGCAATTCCGGGGATCCGTCGACC
DHL_P530_F	TTGTAAAACGACGGCCAGTGAATTCGAGCTCAGCGGTGGCGGTGGCAGTAACGATGGATCCGAAATCGGTACTGGCTTTC
DHL_P531_R	ACTTCGAAGCAGCTCCAGCCTACACCCCGGGTTAACCGGAAATCTCCAGAGTAGACAGCC
DHL_P550_F	GTCACGACGTTGTAAAACGACGGCCAGTGAATTCGAGCTCAGCGGTGGCGGTGGCAGTAA
DHL_P631_R	GCTTTTCGTTCGGGTCTTTGGACAGTTTAGACTGGGTGGACAGGTAGTGGTTATC
DHL_P632_F	GGATAACCACTACCTGTCCACCCAGTCTAAACTGTCCAAAGACCCGAACGAAAAG
DHL_P633_R	TAGGAACTTCGAAGCAGCTCCAGCCTACACCCCGGGTTATTTGTAGAGTTCATCC

Plasmid pDHL940 was constructed by PCR amplifying the HaloTag H7 variant from the pFC14A HaloTag CMV Flexi vector (Promega) using primers DHL_P530_F and DHL_P531_R. The resulting PCR product was inserted into the SacI/XmaI-digested pUC19-flippase recognition target (FRT)-Kan^r^-FRT vector (pDHL19; Paulsson lab plasmid collection) using isothermal assembly (ITA).

Plasmid pDHL1029 was built by PCR amplification of two inserts, which correspond to the first part (amino acids [aa] 2 to 205) and second part (aa 207 to 238) of superfolder GFP, from plasmid pDHL584 using primers DHL_P550_F and DHL_P631_R and primers DHL_P632_F and DHL_P633_R, respectively. Primers DHL632_F and DHL_P631_R contain V206K in their overhangs. The PCR products were then inserted into the SacI/XmaI-digested pUC19-FRT-Kan^r^-FRT vector (pDHL19; Paulsson lab plasmid collection) using ITA.

Plasmid pDHL-HaloSS was constructed by traditional subcloning. The HaloSS sequence was synthesized by Genewiz and cloned into pUC57-Kan^r^. The pUC57-HaloSS-Kan^r^ vector was digested with SacI and XmaI to release the HaloSS fragment, which was then subcloned into SacI/XmaI-digested pDHL584. All constructed plasmids were verified by analytical digestion and DNA sequencing.

The construction of the E. coli strains with translational fusions to DsbA and ClpP at their endogenous loci was performed with the lambda red method (45), as previously described (43). Primers with 50-nucleotide upstream or downstream homology (for DsbA tagging, DsbA_F and DsbA_R; for ClpP tagging, DHL_P182_F and DHL_P183_R) were used to PCR amplify the respective integration cassettes using pDML584 (sfGFP), pDML940 (HaloTag), pDML1029 (monomeric sfGFP [msfGFP]), or pDHL-HaloSS as the template.

### TMR ligand agar plates and crude cell labeling.

For on-plate labeling of E. coli cells, 5 nM HaloTag TMR ligand (catalog no. G825A; Promega, Madison, WI) was added to M63 minimal medium prior to the making of the agar plates. The strains were streaked onto the plates and incubated at 30°C for 24 h. For labeling of live cells, cell cultures after overnight growth at 30°C were mixed with the HaloTag TMR ligand at a final concentration of 5 μM and incubated at 30°C for 15 min. Cells were pelleted by centrifugation and washed twice with phosphate-buffered saline (PBS), resuspended in 1× SDS loading buffer, and subjected to SDS-PAGE analysis. TMR-labeled Halo fusion proteins both on the agar plates and on the SDS-PAGE gel were detected by direct fluorescent scanning using a Typhoon 9400 (setting, 532-nm green laser and a 580-band-pass emission filter).

### Motility plates.

A single colony was stabbed in the middle of an M63 minimal plate containing 0.3% agar. Plates were incubated at 30°C for 48 h.

### Western blotting.

Western blotting was carried out by following a standard protocol. The RpoS expression level was measured in the various *clpP* fusion strains and the Δ*clpPX* strain using a monoclonal anti-RpoS antibody (Neoclone; W0009). The expression of DsbA and its various fusions was detected by using an anti-DsbA antibody (Berkmen lab collection). The secondary antibody, goat anti-rabbit antibody–IRDye 800CW (catalog no. 925-32211), was purchased from Li-Cor Biotechnology (Lincoln, NE). The blot was scanned with an Odyssey infrared imaging system.

### Microscopy.

The respective E. coli strains were grown at 37°C with shaking (250 rpm) to mid-exponential phase in LB medium supplemented with the appropriate antibiotics. The HaloTag TMR ligand (catalog no. G825A; Promega, Madison, WI) was diluted in dimethyl sulfoxide (DMSO) (Sigma-Aldrich) to a final concentration of 500 μM, and ∼5-μl aliquots were stored at −20°C. The HaloTag-expressing E. coli strains and the wild-type strain, which served as a negative control to demonstrate the specificity of the labeling experiment, were processed in parallel and subjected to identical treatments. One milliliter of mid-exponential-phase cells was pelleted (10,000 × *g*, 1 min) and resuspended in 100 μl of a fresh LB medium. The bacterial cells were incubated for 30 min with 5 μM (or 0.5 μM) HaloTag TMR ligand and then washed 5 times with 1 ml of LB medium to remove the free TMR dye. The cells were finally resuspended in 1 ml of M9 medium supplemented with 0.2% (wt/vol) glucose and 10% (vol/vol) LB. Approximately 2 μl of the cell suspension was then spotted onto an agarose pad made with 2% (wt/vol) low-gelling-temperature agarose (catalog no. A9414; Sigma-Aldrich) dissolved in M9 medium with 0.2% (wt/vol) glucose. The cells were allowed to sit on the agar pad for 10 to 15 min prior to microscopy.

Imaging was performed with an inverted microscope (Nikon Ti-E) equipped with either an Orca R2 (Hamamatsu) or a Clara (Andor) interline charge-coupled-device (CCD) camera, a Spectra light engine (Lumencor), and a 100× objective (Nikon). The TMR-stained bacteria were imaged with the green LED and a Cy3 filter cube (TRITC-B; Semrock), whereas GFP-expressing cells were imaged with the blue LED and a GFP filter cube (FITC-2024B; Semrock).

## RESULTS

### Use of the HaloTag in prokaryotes.

**FIG 1:**
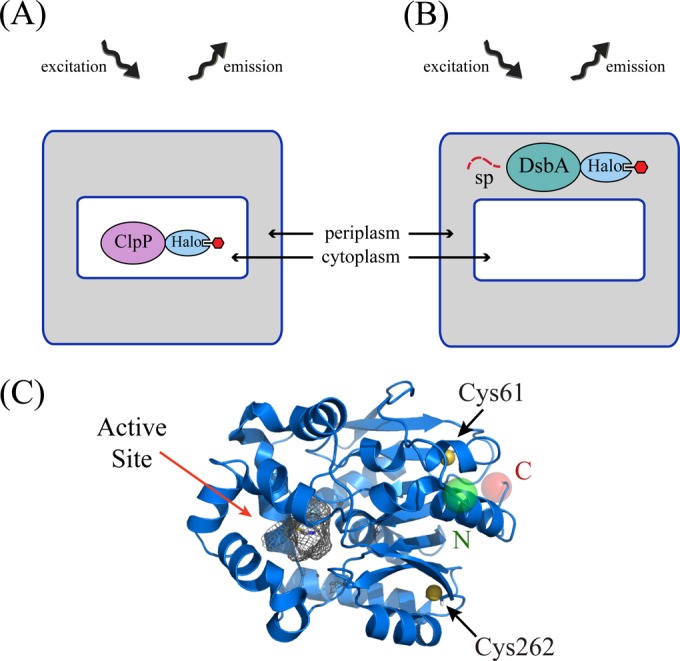
Use and structure of HaloTag in E. coli. C-terminal HaloTag fusions are visualized by capturing the light emission (580 nm) from the TMR ligand after its excitation (550 nm). HaloTag fusions can be visualized either in the cytoplasm, as in the case of the ClpP-Halo fusion (A), or by directing the fusion to the periplasm using a signal sequence (ss), as in the case of the DsbA-Halo fusion (B). (C) The crystal structure of haloalkane dehalogenase (HaloTag) from Rhodococcus (PDB accession no. 1BN7) (65). The active-site groove where the TMR ligand binds, the two cysteines, and the amino (green ball; N) and carboxyl (red ball; C) termini are indicated.

In this study, we applied the HaloTag technology to visualize, for the first time, proteins localized to the cytoplasm and to the periplasm of the model prokaryotic organism E. coli (Table 3, use of various technologies to visualize proteins in E. coli). We used the periplasmic disulfide bond oxidase DsbA and the cytoplasmic protease ClpP as model proteins to test the use of the HaloTag as an aid to visualize periplasmic and cytoplasmic proteins (Fig. 1). DsbA is naturally targeted to the periplasm via its SRP-dependent signal peptide (46), which was also shown to be essential for the efficient secretion of a DsbA-sfGFP fusion to the periplasm (20). A ClpP-SNAP tag fusion has been previously used to illustrate the SNAP tag technology for imaging in chemically fixed (43) and live bacteria (47–49), thus making it an ideal protein to test and compare to the HaloTag technology. Further, the ClpP homo-oligomer is sensitive to fluorescent protein-mediated aggregation (i.e., focus formation) (43), which provides a test for the dimerization/multimerization tendency of the HaloTag in the context of an aggregation-sensitive protein.

**TABLE 3:** Summary of fluorescence and bioluminescence visualizations of proteins in the two compartments of E. coli

Construct	Reference for cytoplasm	Reference(s) for periplasm
GFP/sfGFP	55	20 (ssDsbA)
SNAP tag	43	56–58 (failed)
HaloTag	This work	This work (ssDsbA)
Luciferase	59 (in Synechococcus)	22 (ssOmpA)
Immunofluorescence	60	61

### HaloTag fusion proteins are functional.

The translational fusion of the HaloTag domain to ClpP or DsbA may result in misfolded and nonfunctional proteins. To assess whether the client protein or the HaloTag domain of the fusions was active, we conducted several assays. Cells expressing correctly folded cytoplasmic ClpP-HaloTag or periplasmic DsbA-Halo fusions can be detected by fluorescence after growing the cells in medium containing the HaloTag TMR ligand. The 636-Da TMR ligand is sufficiently small to enter living bacterial cells when supplied exogenously. Thus, a functional HaloTag fusion should covalently bind the TMR ligand and emit light at ∼580 nm when excited with ∼550 nm light. Various E. coli strains producing either periplasmic DsbA-Halo or cytoplasmic ClpP-Halo fusions were streaked on minimal M63 plates containing 5 nM TMR ligand, and we imaged the bacterial colonies on plates using a Typhoon gel imager. Only the cells producing the HaloTag fusions emitted light at 580 nm. A 3-fold-lower fluorescent signal was observed for the strain producing DsbA tagged with a mutant Halo_C61S C262S_ tag protein lacking cysteines (coined HaloSS). We created this mutant to test whether a cysteine-free version of the HaloTag would show improved targeting to the periplasmic space and would result in increased periplasmic labeling.

**FIG 2:**
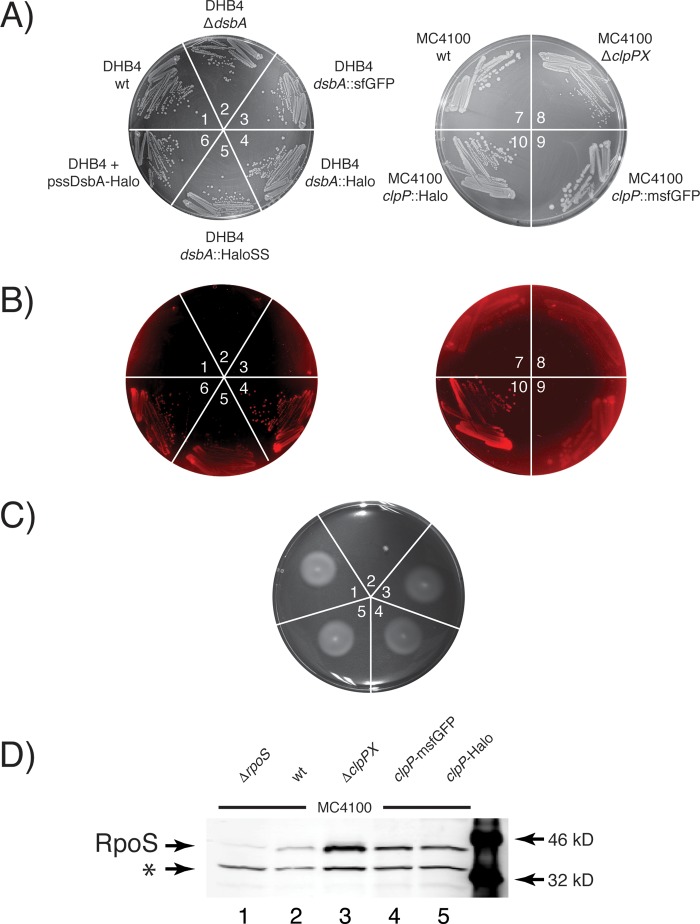
HaloTag and GFP fusions are functional. (A to C) MB10 (strain 1), MB68 (strain 2), MB3106 (strain 3), MB3104 (strain 4), MB3105 (strain 5), MB3769 (strain 6), MB3123 (strain 7), MB3716 (strain 8), MB3717 (strain 9), and MB3128 (strain 10) were streaked out on minimal M63 plates containing 5 nM TMR ligand. Pictures of the plates were taken with bright-field illumination (A) and fluorescent emission (B) using a 532-nm laser for excitation. (C) E. coli cultures were spotted on low-agar M63 motility plates, which were then incubated at 30°C for 48 h. (D) Immunoblot of RpoS in cells lacking a chromosomal copy of *rpoS* (MB4156, lane 1), wild-type cells (MB3123, lane 2), cells lacking a chromosomal copy of *clpPX* (DHL708, lane 3), and cells whose chromosomal copy of *clpP* has been replaced with either *clpP*-msfGFP (MB3717, lane 4) or *clpP*-HaloTag (MB3128, lane 5). Thirty-eight-kilodalton RpoS and an unspecific cross-reacting band (*) are indicated.

The ability to easily detect the correct expression of the HaloTag fusion proteins within living bacterial cells allowed us to discern whether transformants are correctly expressing the HaloTag fusions or not. This was demonstrated by mixing cell cultures that had either an empty vector (MB10) or a HaloTag fusion (MB3104) in a 1:1 ratio (using an optical density at 600 nm) and plating the cells on minimal M63 plates containing 5 nM TMR ligand (see Fig. S1 in the supplemental material). Individual colonies of cells that expressed the HaloTag fusion could be easily detected by illuminating the plate with light at 532 nm using the green laser of the Typhon 9400 (GE Healthcare) gel imager (Fig. 2A and B).

To assess whether the periplasmic disulfide bond oxidase DsbA is active, we conducted a motility assay. E. coli cells lacking a functional copy of *dsbA* are nonmotile, as FlgI, one of the components of the flagellum, requires a disulfide bond for its stability (50). Consequently, E. coli cells with the chromosomal copy of *dsbA* replaced by the *dsbA*-Halo translational fusion should be motile only if a functional DsbA-Halo fusion is exported to the periplasm. As expected, chromosomal deletion of *dsbA* results in a nonmotile strain when it is grown on low-concentration agar plates (0.75%, wt/vol, agar), while cells expressing a single copy of *dsbA*-Halo, *dsbA*-HaloSS, or *dsbA*-sfGFP were all motile (Fig. 2C).

We then assessed the functionality of the cytoplasmic ClpP-HaloTag fusion by measuring the protein levels of RpoS, which is the master transcriptional regulator of the stress response and stationary phase and is a proteolytic substrate of ClpP (51). Immunoblot analysis of RpoS, using a monoclonal anti-RpoS antibody, indicated increased amounts of RpoS in a strain that lacked the chromosomal copy of *clpP* (Fig. 2D, lane 3), which confirms the role of ClpP in degrading RpoS (48). The chromosomal replacement of *clpP* with either the *clpP*-msfGFP or *clpP*-Halo translational fusion lowered the levels of RpoS, albeit not completely back to wt levels (Fig. 2D, lanes 4 to 5). These results indicate that the ClpP-Halo and ClpP-msfGFP fusions are at least partially proteolytically functional.

Taken together, our data suggest that both the DsbA (periplasm) and ClpP (cytoplasm) HaloTag fusions are functional and can readily be detected in cells grown on medium containing the HaloTag TMR ligand.

### Expression of the HaloTag fusions.

Fusing the 34-kDa HaloTag to DsbA may impair its secretion and/or the redox state of the active-site cysteines in DsbA. We therefore further characterized the expression levels of the DsbA-Halo fusions and the redox state of their cysteines by AMS (4-acetamido-4′-maleimidylstilbene-2,2′-disulfonic acid) alkylation followed by immunoblot analysis using an anti-DsbA antibody. AMS alkylates free cysteines, adding 0.5 kDa. Therefore, alkylation of reduced and nonreduced samples leads to different electrophoretic sizes. DHB4 cells expressing the HaloTag domain fused to the signal sequence (ss) of DsbA (ssDsbA-Halo) from a plasmid or DHB4 cells whose genomic copy of *dsbA* was replaced with *dsbA*-sfGFP, *dsbA*-Halo, or *dsbA*-HaloSS were grown in minimal medium and subjected to trichloroacetic acid (TCA) precipitation prior to being immunoblotted. The fully denatured cell lysates were resuspended in either buffer or buffer with AMS or were fully reduced with dithiothreitol (DTT) followed by AMS alkylation. AMS is an alkylating agent that forms covalent bonds with any thiol group within reduced cysteines, adding 500 Da per cysteine to the molecular mass of the protein. Thus, a DsbA-Halo fusion with altered redox state cysteines should migrate differently than the untagged (control) DsbA protein.

**FIG 3:**
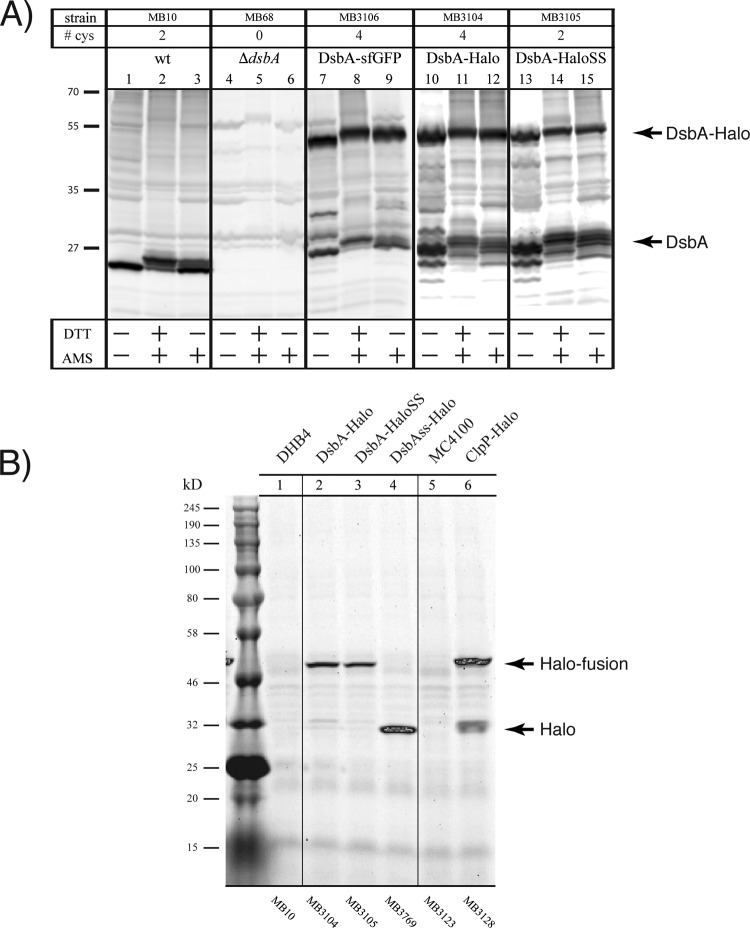
Analysis of the HaloTag fusions. (A) Production levels and redox states of DsbA fusions by immunoblot analysis. Various E. coli strains were grown in minimal M63 medium and the total proteome of the cells was denatured using TCA. The samples were resuspended in buffer (lanes 1, 4, 7, 10, and 13) or in buffer containing AMS (lanes 3, 6, 9, 12, and 15), or they were reduced by DTT and then alkylated by AMS (lanes 2, 5, 8, 11, and 14). The samples were probed with an anti-DsbA antibody and subjected to immunoblot analysis. Protein bands corresponding to the DsbA-Halo fusions and untagged DsbA, along with the number of cysteines (cys) per protein, are indicated. (B) In-gel labeling of the HaloTag fusions. Various E. coli strains with the empty vector (lane 1 and 5) or producing a DsbA-Halo fusion (lane 2), DsbA fused to a HaloTag variant lacking cysteines (lane 3), the HaloTag with the *dsbA* signal sequence (lane 4), or the ClpP HaloTag fusion (lane 6) were grown in minimal M63 medium to late-stationary phase, incubated with the HaloTag TMR ligand, and subjected to in-gel TMR visualization.

Immunoblot analysis followed by AMS alkylation revealed, as in previous publications (52), that the majority of DsbA is in the oxidized state in wt cells (Fig. 3A, lane 1 versus lane 3). The observed protein bands are specific to DsbA, as none of the major bands detected were observed in Δ*dsbA* cells (Fig. 3A, lanes 4 to 6). Fusions of sfGFP (Fig. 3A, lanes 7 to 9) and the HaloTag (Fig. 3A, lanes 10 to 12) to DsbA were detected at the expected sizes. Since the cysteines present in the HaloTag are susceptible to oxidation in the periplasm, we also engineered a DsbA-Halo fusion, which has the two cysteines in the HaloTag (i.e., Cys61 and Cys261) mutagenized to serine residues (HaloSS). Although this construct resulted in a lower fluorescent signal when the cells were grown in TMR ligand-containing medium (Fig. 2B, sector 5), the expression level and the redox state of the HaloSS fusion were comparable to those of the fusion with the normal HaloTag, indicating that the lower fluorescence signal is not due to poor expression or altered redox state. We speculate that the HaloSS fusion is less reactive and has a lower labeling efficiency when incubated with the TMR ligand. Alternatively, the TMR-labeled HaloSS protein may also be less bright than the TMR-labeled HaloTag protein, because the two cysteine mutations alter the chemical environment and, hence, the fluorescence intensity of the bound TMR molecule. However, in all cases, proteolytically cleaved DsbA products were detected around the expected size of DsbA. This was also observed previously with a MalE-sfGFP fusion (20).

It is also possible to detect functional HaloTag fusions by incubating the cell culture with the TMR ligand, followed by cell lysis and SDS-PAGE analysis. During the incubation period, the TMR molecules permeate through the cell membrane and covalently bind to any functional HaloTag domain. The TMR molecules remain covalently bound during the SDS-PAGE analysis. This method permits us to probe the functionality of the HaloTag protein in the context of the protein fusions and detect any proteolytically degraded, yet functional, HaloTag proteins. Cells expressing the DsbA-Halo, DsbA-HaloSS, ssDsbA-Halo, or ClpP-HaloTag fusion were incubated with the TMR ligand, and the total lysates were subjected to SDS-PAGE (Fig. 3B). All of the constructs were detected at the expected sizes, and no proteolytically cleaved HaloTag domain was observed for the DsbA fusion proteins (Fig. 3B, lanes 2 and 3). However, a small amount of proteolytically cleaved HaloTag protein was observed in the case of the ClpP-HaloTag fusion, which was expressed in the cytoplasm (Fig. 3B, lane 6). This might be due to an increased sensitivity of the ClpP-HaloTag fusion to proteolysis and the larger amounts of proteases present in the cytoplasm than in the periplasm (Fig. 3B, lanes 2 and 3 versus lane 6).

### Microscopic visualization of the HaloTag fusions.

**FIG 4:**
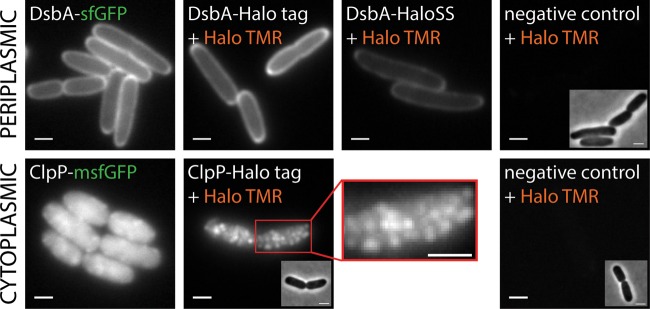
Visualization of periplasmic and cytoplasmic HaloTag fusion proteins in live E. coli cells by fluorescence microscopy. The HaloTag is functional in the periplasm (upper row) and cytoplasm (lower row). Live E. coli cells expressing the *dsbA*-Halo or *dsbA*-HaloSS mutant were labeled with the TMR ligand and visualized by epifluorescence microscopy. Individual ClpP-HaloTag particles were visualized in fixed E. coli cells (lower row, middle) by labeling live cells with the TMR ligand followed by chemical fixation. The image corresponds to a maximum projection of a z-stack (11 slices with 100-nm spacing). The red box shows a closeup. The ClpP particles are highly mobile, which is consistent with the uniform fluorescent signal of ClpP-msfGFP in live cells (lower row, left). ClpP-msfGFP does not form artificial foci due to fluorescent protein dimerization. The ClpP-HaloTag fusion still shows some weak residual artifactual foci formation (see Fig. S2 in the supplemental material), indicating that the HaloTag is not truly monomeric. Scale bars, 1 μm.

Our results indicate that we can express and detect functional HaloTag fusions both in the cytoplasmic and in the periplasmic compartment. In order to evaluate whether the HaloTag fusions are expressed in the correct compartments, microscopic analysis of cells expressing genomic constructs of ClpP-Halo, ClpP-msfGFP, DsbA-Halo, DsbA-HaloSS, or DsbA-sfGFP tags was conducted. Cells were grown to mid-log phase, labeled with the TMR ligand, and then imaged on agarose pads. We imaged the ClpP-msfGFP and DsbA-sfGFP strains in parallel as positive controls for protein localization (Fig. 4). Both the DsbA-sfGFP and DsbA-Halo constructs displayed a largely peripheral fluorescent signal with some weak polar accumulation. The peripheral localization of the DsbA fusions was essentially identical to that in a previous study (20). The Halo TMR ligand specifically reacts with the HaloTag and does not label other endogenous E. coli proteins. Nonspecific labeling was very low. While cells expressing the DsbA-HaloSS fusion displayed a much weaker signal, the localization pattern was very similar to that with the DsbA-Halo fusion. The weaker signal observed with the DsbA-HaloSS fusion by microscopy (Fig. 4; see also Movie S5 in the supplemental material) is in agreement with the weaker fluorescent signal of the DsbA-HaloSS microcolonies grown on the TMR ligand agar plates (Fig. 2B, sector 5). The strong peripheral labeling of the DsbA-Halo, DsbA-HaloSS, and DsbA-sfGFP fusions indicates that the HaloTag can indeed be used to visualize periplasmic proteins in living E. coli cells (Fig. 4; see also Movies S1, S2, and S5 in the supplemental material). Furthermore, time-lapse movies of the same constructs were acquired, and the movement of the DsbA-Halo molecules could be observed (Fig. 4; see also Movie S1 in the supplemental material). The movements of the dim DsbA-Halo foci were similar to those in previous time-lapse movies of other periplasmic proteins labeled with GFP (see Movies S1 and S5 versus Movie S2 in the supplemental material) (20).

Cytoplasmic labeling of the ClpP-Halo fusion with the TMR ligand allowed the visualization of individual ClpP oligomers in live (Fig. 4; see also Movie S3 in the supplemental material) and fixed (Fig. 4; see also Z-stack Movie S4 in the supplemental material) E. coli cells. The observed localization pattern and movement of ClpP are consistent with previous findings (43). ClpP-Halo fusion did result in weak artifactual foci when fused to an aggregation-prone protein, such as the homo-oligomer ClpP (see Fig. S2 in the supplemental material). However, HaloTag is more monomeric than the sticky fluorescent proteins, such as normal superfolder GFP or venus YFP (without the A206K mutation) (43). To detect and avoid localization artifacts, we recommend comparing the localization pattern of a protein fusion with the HaloTag to that of the corresponding protein fusion with monomeric GFP (e.g., msfGFP or mEGFP) (see Fig. S2 in the supplemental material).

To our knowledge, our study is the first example of the use of the HaloTag both in the cytoplasm and in the periplasm of a prokaryotic cell.

## DISCUSSION

In this paper, we demonstrate the use of the HaloTag as a translational fusion to visualize the localization of proteins in the model prokaryote E. coli. We achieved this by fusing the HaloTag to the carboxyl terminus of the periplasmic disulfide bond oxidase DsbA and to that of the cytoplasmic protease ClpP. We confirmed that full-length fusions of the expected molecular weight are expressed. We were able to detect expression of functional HaloTag fusion proteins *in vivo* in colonies grown on agar plates and in cells grown in liquid culture (analyzed by SDS-PAGE). Microscopic analysis further revealed the peripheral localization of the DsbA-Halo fusion protein in the periplasm and multiple diffraction-limited spots in the cytoplasm with the ClpP-Halo fusion, which are reminiscent of earlier studies using fluorescent proteins and immunofluorescence (43). Our data are in strong agreement with previous observations and confirm the validity of the use of the HaloTag in prokaryotes.

The use of the HaloTag to visualize proteins not only adds a new molecular tool to investigate proteins in E. coli but also opens the path to a myriad of new applications. An immediate application would be the ability to visualize proteins together with other fluorescent proteins, such as GFP. As discussed, HaloTag fusions can result in weak artifactual focus formation when fused to an aggregation-prone protein, like the homo-oligomer ClpP (see Fig. S2 in the supplemental material). We recommend performing appropriate control experiments using a fusion with monomeric GFP or conducting immunofluorescence against the untagged protein of interest. This study opens up the possibility of using the HaloTag and the TMR ligand in combination with a monomeric GFP to visualize ClpX (or ClpA) and ClpP in the same cell to study protease dynamics, colocalization, and protease assembly. Using superresolution imaging or underexpression of the protease might allow investigations about whether the ClpP oligomer is always associated *in vivo* with the ClpX or ClpA chaperones (i.e., ClpXP and ClpAP, respectively) and, for example, how the proteases segregate at cell division (66).

Unlike autofluorescent tags, such as GFP and its variants, a single HaloTag fusion can also be labeled with multiple fluorescent dyes or otherwise functionalized ligands in a single cell (30). Since the biophysical properties of the HaloTag ligand can be modulated by various chemistries, ligands could be modified to the needs and constraints imposed by the experiment. For example, the ability to label the HaloTag *in situ* permits the use of a blocking ligand, thus enabling visual pulse-chase experiments (54). Therefore, one can follow the movement and turnover of batch-labeled proteins in single cells over time by microscopy. Further applications, such as screening DNA libraries for functional expression of fusion proteins and even testing a variety of ligands *in vivo*, might be achieved.

## Supplementary Material

Supplemental material
