# Supplementary material for: Visualization of Periplasmic and Cytoplasmic Proteins with a Self-Labeling Protein Tag
Source: J Bacteriol. 2016 Mar 17;198(7):1035–43. doi: 10.1128/JB.00864-15 (PMC4800872; doi:10.1128/JB.00864-15)
Supplement: Supplemental material [file supp_198_7_1035__index.html]

Visualization of Periplasmic and Cytoplasmic Proteins with a Self-Labeling Protein Tag — Supplemental material 

# Visualization of Periplasmic and Cytoplasmic Proteins with a Self-Labeling Protein Tag

## Supplemental material

- Supplemental file 1 -

  Movie S1 (*E. coli* cells producing DsbA-Halo fusion)

  MOV, 1.1M
- Supplemental file 2 -

  Movie S2 (*E. coli* cells producing DsbA-sfGFP fusion)

  MOV, 1.2M
- Supplemental file 3 -

  Movie S3 (*E. coli* cells producing ClpP-Halo fusion)

  MOV, 5.2M
- Supplemental file 4 -

  Movie S4 (*E. coli* cell producing ClpP-Halo fusion)

  MOV, 2.7M
- Supplemental file 5 -

  Movie S5 (*E. coli* cells producing DsbA-HaloSS fusion)

  MOV, 2.1M
- Supplemental file 6 -

  Fig. S1 (Halo fusion screening of functional transformants) and S2 (*E. coli* cells expressing ClpP fusions) and legends to Movies S1 to S5

  PDF, 12M
