# Supplementary material for: Visualization of Periplasmic and Cytoplasmic Proteins with a Self-Labeling Protein Tag
Source: J Bacteriol. 2016 Mar 17;198(7):1035–43. doi: 10.1128/JB.00864-15 (PMC4800872; doi:10.1128/JB.00864-15)
Supplement: Supplemental material [file JB.00864-15_zjb999093973so6.pdf]

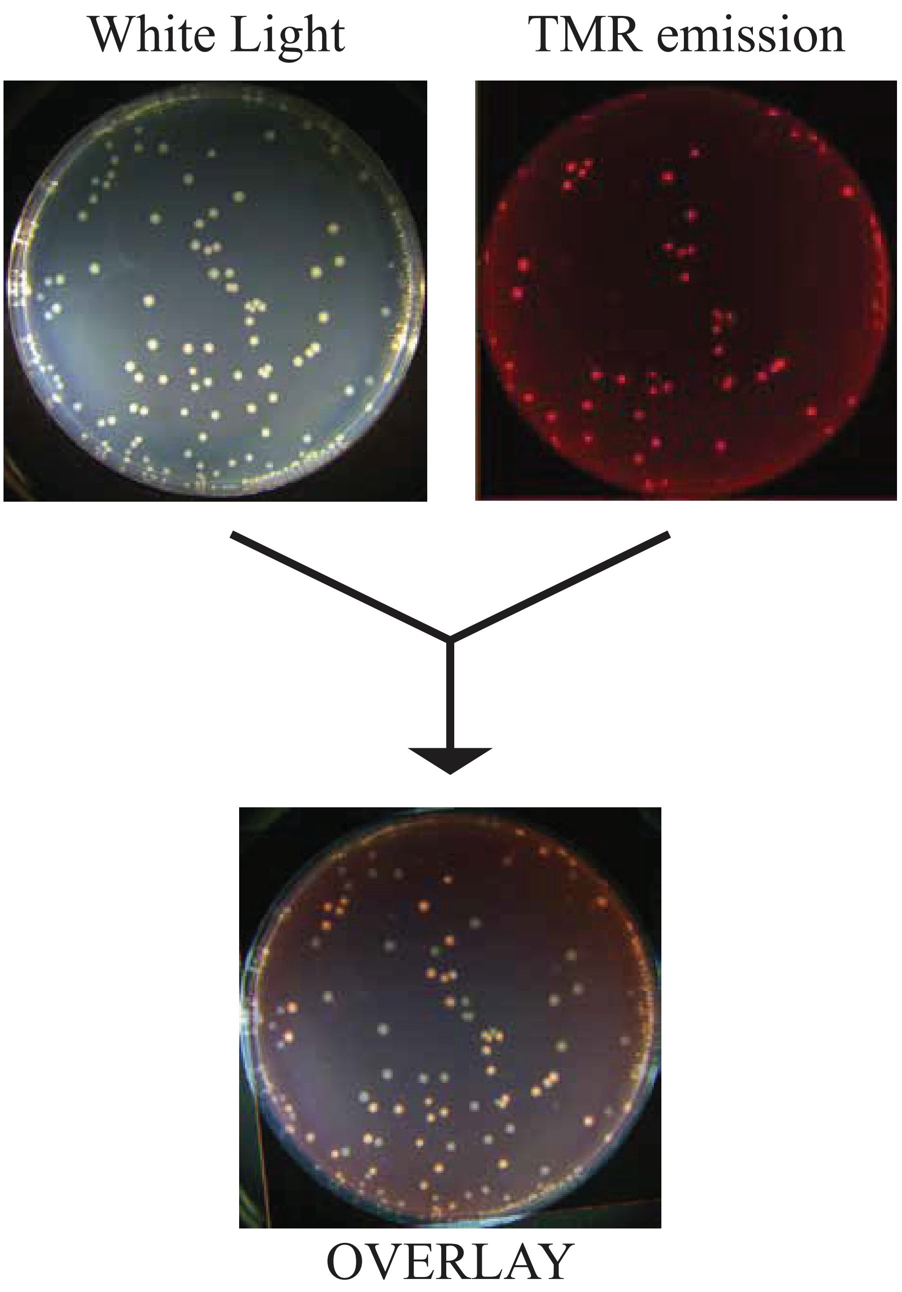

Supplementary Figure 1: Halo fusions can be used to screen functional transformants. Mixed cell cultures having either an empty vector (MB10) or DsbA-Halo fusions (MB3104) were mixed 1:1 (standardized using OD600) and plated on minimal M63 plates containing 50 nM TMR ligand. Pictures of the agar plate were taken with either white light or with yellow light (for TMR excitation) and overlaid.

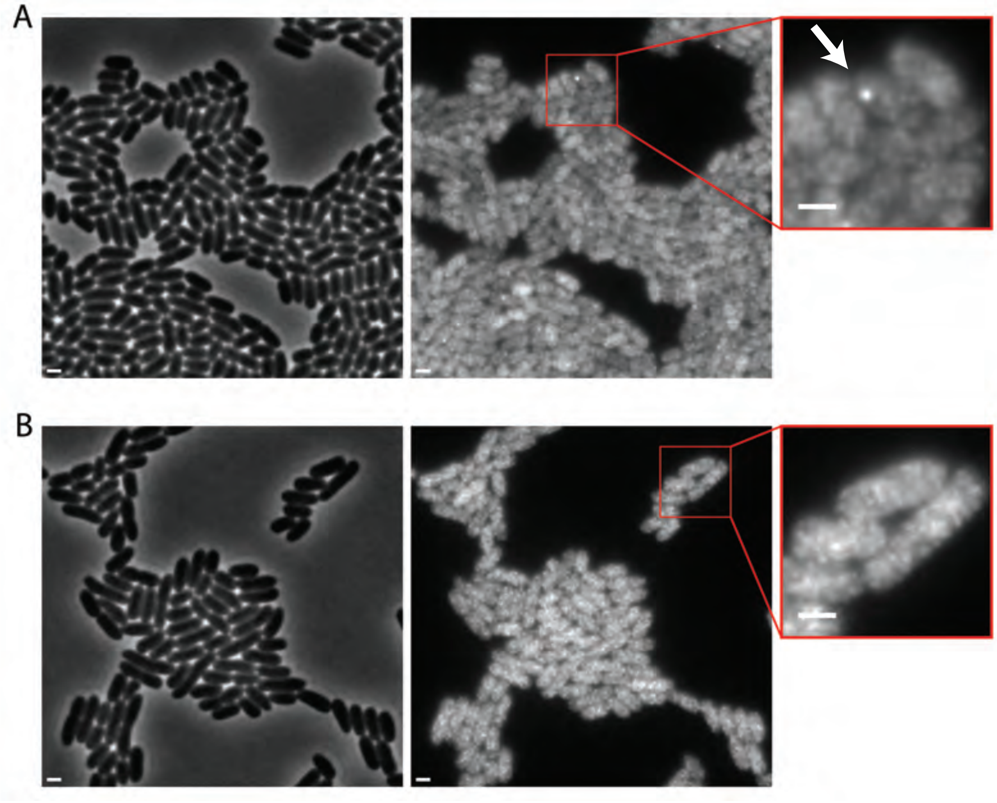

Supplementary Figure 2: Imaging of chemically fixed *E. coli* cells that express the ClpP-HaloTag fusion (A) or the ClpP-msfGFP fusion (B). The ClpP-HaloTag fusion shows a weak tendency to form foci (arrow), which is not observed with the ClpP-msfGFP fusion. A single cell with a ClpP-HaloTag focus is shown in the close-up (upper row, right). The HaloTag was labeled with the TMR ligand. Individual ClpP oligomers are discernable with the ClpP-HaloTag and the ClpP-msfGFP fusion. Cells have approximately 20-50 ClpP oligomers. Phase images are shown on the left and fluorescence images with a close-up are shown on the right. Scale bar (white) is 1 μm.

Supplemental Movie S1: Live-cell fluorescence microscopy of *E. coli* cells producing DsbA-Halo fusion labeled with the TMR dye. The cells were labeled with 0.5 μM TMR ligand, washed extensively, and imaged on an agar pad. The exposure time is 200 ms. Scale bar (white) is 1 μm.

Supplemental Movie S2: Live-cell fluorescence microscopy of *E. coli* cells producing DsbA-sfGFP fusion. The cells were imaged on an agar pad. The exposure time is 200 ms. Scale bar (white) is 1 μm.

Supplemental Movie S3: Live-cell fluorescence microscopy of *E. coli* cells producing ClpP-Halo fusion labeled with the TMR dye. The cells were labeled with 0.5 μM TMR ligand, washed extensively, and then imaged on a bare coverslip. The exposure time is 200 ms. Scale bar (white) is 1 μm.

Supplemental Movie S4: Z-stack of an *E. coli* cell producing ClpP-Halo fusion labeled with the TMR dye followed by chemical fixation. Individual ClpP oligomers are clearly visible as diffraction-limited spots, although many spots overlap. The movie shows 21 planes and the spacing between individual planes is 100 μm. The exposure time is 2 s for each plane of the z-stack. Scale bar (white) is 1 μm.

Supplemental Movie S5: Live-cell fluorescence microscopy of *E. coli* cells producing DsbA-HaloSS fusion labeled with the TMR dye. The cells were labeled with 0.5 μM TMR ligand, washed extensively, and then imaged on an agar pad. The exposure time is 2 s. Scale bar (white) is 1 μm.
